# Evaluating the relaxation effects of Shikuwasa (Citrus depressa Hayata) essential oil inhalation in young female adults: Study protocol for a randomised controlled trial

**DOI:** 10.1016/j.conctc.2024.101342

**Published:** 2024-07-26

**Authors:** Fumitake Yamaguchi, Naoki Yoshinaga, Miho Kuroki, Rie Nakasone, Hisanori Kenmotsu, Toshio Ueno, Yukihiro Yada, Michikazu Nakai, Yasuji Arimura

**Affiliations:** a School of Nursing, Faculty of Medicine, University of Miyazaki, 5200 Kihara, Kiyotake, Miyazaki City, Miyazaki, 889-1692, Japan; b Department of Health Care Research, Organization for Promotion of Research and Industry-Academic Regional Collaboration, University of Miyazaki (Kiyotake Branch), Miyazaki City, Miyazaki, 889-1692, Japan; c Material Research & Development Division, Ogawa & Co., Ltd., 7 Oaza-Hoshinosato, Amimachi, Inashiki-gun, Ibaraki, 300-0326, Japan; d Program in Human Biology, School of Integrative and Global Majors, Graduate School of Comprehensive Human Sciences, University of Tsukuba, 1-1-1 Tennodai, Tsukuba, Ibaraki, 305-8577, Japan; e Clinical Research Support Center, University of Miyazaki Hospital, Faculty of Medicine, University of Miyazaki, 5200 Kihara, Kiyotake, Miyazaki City, Miyazaki, 889-1692, Japan

**Keywords:** Aromatherapy, Citrus, Clinical trial protocol, Essential oils, Japan, Relaxation, Randomised controlled trial, Shikuwasa

## Abstract

**Introduction:**

The essential oil of Shikuwasa (*Citrus depressa* Hayata) primarily contains limonene and γ-terpinene, which have potential applications in stress management and relaxation. However, the psychological or physiological relaxation effects of Shikuwasa essential oil on humans are still unknown. This study aims to investigate the short-term relaxation effects of Shikuwasa essential oil, one of the less-studied varieties, compared to inhaling odour-free air in young female adults.

**Methods:**

and analysis: This study is a two-arm, parallel-group, open-label, randomised controlled superiority trial. Forty young female adults will be assigned with a 1:1 allocation ratio to either the Shikuwasa essential oil inhalation group or the odour-free air inhalation group. The primary outcome measure will be subjective tense arousal (subscale of the Japanese version of the University of Wales Institute of Science and Technology Mood Adjective Checklist). Secondary outcomes include objective measures: miosis rate and peripheral skin temperature for evaluating autonomic nervous activity, and cerebral blood flow (assessed using near-infrared spectroscopy) for evaluating central nervous activity. Since these objective outcome measures cannot be performed at the same time, we divide our experiment into three phases and participants will inhale sample vials for 2 min in each experiment. We will also evaluate individual preferences/impressions regarding inhaled samples and any adverse events.

**Ethics and dissemination:**

The study protocol has been reviewed and approved by the Research Ethics Committee of the Faculty of Medicine, University of Miyazaki (reference no: I-0074). The findings of this study will be disseminated to academic and professional audiences via publications in peer-reviewed journals and presentations at academic conferences, and to the broader public via public talks and media/press releases. All study findings, whether negative or positive, will be reported.

**Trial registration:**

UMIN Clinical Trials Registry (UMIN-CTR), UMIN000053914. Prospectively registered on March 20, 2024.

## Highlights

• This is the first randomised cotrolled trial to evaluate the short-term relaxation effects of Shikuwasa essential oil in humans.
• This study employs various objective outcomes to speculate on psychological and physiological states, supplemented by subjective outcomes.
• Limitations: Dividing the procedure into three phases; the sample restricted to young Japanese females; use of a non-active control condition.

## 1 Introduction

Over the decades, traditional and complementary medicine, including the use of natural plant essential oils, has gained attention globally for promoting people's health and well-being [[1], [2], [3]]. The use of essential oils has been widely studied and findings suggest that some of their benefits can be attributed to their anti-inflammatory, antiviral, antibacterial, antiseptic, and antifungal properties [4]. The main pathways for the absorption of essential oils into the body include inhalation, skin absorption, and ingestion, with inhalation via the olfactory system (through the respiratory system or olfactory nerves) being the fastest and easiest [5].

Citrus essential oils have the largest market among similar consumer products [6], and have been studied for their antimicrobial and antioxidant properties [7,8]. Citrus essential oils are fragrant volatile molecules and include monoterpenes and their derivatives, aldehydes, ketones, esters, alcohols, and bioactive compounds such as limonene, β-pinene, and γ-terpinene [9,10]. Several citrus essential oils, such as orange [11] and bitter orange [12,13], have been found to exhibit anxiolytic, antidepressant, anticonvulsant, analgesic, and sedative effects. In addition, citrus essential oils are obtained mainly from citrus peels by cold pressing or distillation, and also from citrus juice by-products such as pulp and pomace. This is environmentally friendly because the peel, which is produced and disposed of in large quantities around the world, can be effectively utilised in value-added products [14].

While there is extensive research on Western citrus essential oils like orange, grapefruit, lemon, and lime [15,16], recent studies have highlighted the potential of Japanese citrus varieties, such as Yuzu (*Citrus junos*) and Iyokan (*Citrus iyo*), in inducing calming effects by modulating autonomic nervous system activity, promoting parasympathetic over sympathetic activation [17]. This property is expected to facilitate mental and physical relaxation. Shikuwasa (*Citrus depressa* Hayata), a small citrus fruit variety well-known in the subtropical regions of Northeast Asia, especially in Okinawa and Kagoshima Prefectures of Japan, is recognized for its tartness, subtle sweetness, and distinctive aroma [18]. The essential oil of Shikuwasa primarily contains limonene and γ-terpinene and shares volatile components similar to Yuzu; but the relative proportions of limonene and γ-terpinene in Shikuwasa are distinct from those in other common citrus cultivars (e.g. grapefruit, orange, and lime), imparting superior and unique aroma characteristics to Shikuwasa [9,19]. A previous small-sized study (having nine healthy female adults) reported that Shikuwasa essential oil and/or its two predominant aroma components (limonene and γ-terpinene) reduced participants’ work errors during visual display tasks, and reduced their stress after completing the task, as determined by the increase in electrocardiogram R-R interval variability and the reduction of Beta wave activity in the electroencephalogram [20]. However, to the best of our knowledge, there is no study investigating the psychological or physiological effects of Shikuwasa essential oil on humans through a randomised controlled study, regardless of the route of administration.

Thus, this study aims to explore the short-term relaxation effects of Shikuwasa essential oil, which is one of the less-studied varieties, through a randomised controlled experimental study design. In this study, we will specifically target young female adults because 1) many studies reported that sex/gender and age are important determinants of smelling ability (incl. odour detection, discrimination, and identification) [21,22], and 2) the market for essential oils is overwhelmingly female [[23], [24], [25], [26]].

## 2 Objectives

The overall aim of this study is to examine the short-term relaxation effects of Shikuwasa essential oil inhalation compared to odour-free air inhalation in young female adults through a randomised controlled trial. More specifically, the primary objective is to determine if a 2-min Shikuwasa essential oil inhalation may reduce subjective tense arousal (subscale of the Japanese version of the University of Wales Institute of Science and Technology Mood Adjective Checklist [JUMACL] [27,28]) compared with 2-min odour-free air inhalation. Our secondary objectives are to determine the short-term effects of Shikuwasa essential oil inhalation compared to odour-free air inhalation on objective outcome measures (miosis rate, peripheral skin temperature, and cerebral blood flow). We will also evaluate individual preferences/impressions regarding inhaled samples and any adverse events.

## 3 Methods

### 3.1 Trial design

This study is a two-arm, parallel-group, open-label randomised controlled superiority trial, comparing the Shikuwasa essential oil inhalation group (experimental group) to the odour-free air inhalation group (control group). Participants will be randomly assigned to either the experimental or control group at a ratio of 1:1. This study protocol was designed in accordance with the Standard Protocol Items: Recommendations for Interventional Trials (SPIRIT) guidelines [29].

### 3.2 Study setting

Healthy young female adults will be recruited from the University of Miyazaki and the local community (Miyazaki prefecture), and the experiment will be conducted at the Faculty of Medicine, University of Miyazaki, Miyazaki, Japan. The study office, which is in the Department of Health Care Research, Organization for Promotion of Research and Industry-Academic Regional Collaboration, University of Miyazaki (Kiyotake Branch), will manage and conduct the study.

### 3.3 Recruitment

Participants will be recruited from the University of Miyazaki and the local community within Miyazaki prefecture, via the student platform (University's portal site), and the study office's (Department of Health Care Research's) website and social media (e.g. Facebook). Recruitment announcements will also be posted at the university's healthcare events and other events.

### 3.4 Eligibility criteria

The inclusion criteria for participants are:

- Female aged 18–39 years at the time of obtaining written informed consent.
- Not being averse to the smell of citrus fruits, including Shikuwasa.
- Expressing a willingness to participate in the study and providing voluntary consent by signing the informed consent form.

The exclusion criteria for participants are:

- Having a history of or currently suffering from clinically problematic respiratory, gastrointestinal, hepatobiliary, haematological, renal, endocrine, cardiovascular, dermatological, or mental disorders.
- Having a history of serious trauma or surgery within 12 weeks prior to the study.
- Being sensitive to cold even in summer.
- Unable to detect odours.
- Having a history of or suspected allergy to foods (especially citrus fruits) or drugs requiring some kind of medical treatment.
- Regularly taking psychotropic drugs (e.g. antidepressants, anxiolytics, or hypnotics)
- Heavy smoker (21 or more cigarettes per day), heavy drinker (regularly drinking more than 60g pure alcohol per day), or having or suspected to have alcohol/substance misuse problems.
- Regularly taking medicines, eating foods for specified health use, foods with function claims, or health food products that may affect the study results.
- Being or possibly being pregnant or breastfeeding.
- Have participated in other clinical trials within 4 weeks prior to the study.
- Identifications of additional reasons, as deemed by study investigators, rendering the participant unsuitable for inclusion in the study.

### 3.5 Allocation

#### 3.5.1 Sequence generation

Participants will be randomly assigned to either the intervention or control group at a ratio of 1:1, with the assignments made by the Viedoc™. Randomisation will be stratified by age (≥25 years or less) and baseline tense arousal scores of JUMACL (≥17 or less). A minimization algorithm will be used to ensure a balance between groups.

#### 3.5.2 Concealment mechanism

The random allocation will be carried out by the Viedoc™, an internet-based central randomisation system. Allocation concealment will be ensured, as the randomisation system will not release the randomisation code until the participant has been recruited into the study and the baseline assessment has been completed.

#### 3.5.3 Implementation

The allocation sequence will be generated before the start of the study by one of the co-investigators (specialist administrative staff) with the cooperation of the Data Management Advisor. Once an eligible participant has given consent to study participation and completed the baseline assessment, the principal investigator or one of the co-investigators (nurse) will access the Viedoc™ and enter the necessary baseline information for randomisation. The random allocation to either group will be centrally generated. The study's principal investigator or one of the co-investigators (nurse) at the study office will then inform the participant about the group allocation.

#### 3.5.4 Blinding and procedure for unblinding

Given the nature of interventions, it is not possible to blind participants or study investigators to participant allocation. Given this is an open-label study, procedures for unblinding are not applicable.

### 3.6 Intervention and comparator

#### 3.6.1 Intervention

The intervention for this study is Shikuwasa essential oil inhalation. Shikuwasa essential oil is prepared from the peel of Okinawa Shikuwasa using a steam distillation method. The essential oil samples used in this study will be provided by Ogawa & Co., Ltd. (Tokyo, Japan). The inhalation method is based on previous studies using Yuzu and Iyokan essential oils [17]. Cotton is soaked in 0.5 μL of Shikuwasa essential oil, and placed on the bottom of an amber vial (opening diameter: 35 mm, height: 73 mm, volume: 50 mL). The lid of the vial is closed and left to stand for 30 min at 25 °C, and the headspace of the vial is filled with odour. After equilibration, the lid is opened and immediately placed under the nose of the study participant. Participants will be instructed to keep the distance between the vial opening and the nose approximately 10 mm, sit comfortably in an armchair during the experiment, and inhale the opening of the sample vial for 2 min with natural breathing in each experiment (see the “Participant timeline” for overall experiment procedures).

#### 3.6.2 Comparator

The comparator for this study is the inhalation of odour-free air (non-active control condition).

Participants assigned to the odour-free air inhalation group will inhale odour-free air in exactly the same manner as those who inhale Shikuwasa essential oil: using the same kind of vial, keeping the distance between the nose and the vial opening approximately 10 mm, sitting comfortably in an armchair during the experiment, and inhaling the sample for 2 min with natural breathing in each experiment. This control condition helps to differentiate the specific effects of the Shikuwasa essential oil from other non-specific effects (e.g. breathing) within the experiment procedures.

#### 3.6.3 Criteria for discontinuing or modifying allocated interventions

Regardless of whether it is related to the inhalation if any adverse event occurs, the inhalation will be stopped immediately. In addition to adverse events, inhalation should also be stopped if the participant complains that the essential oil cannot be inhaled due to its unacceptable fragrance or other reasons. No re-inhalation after the discontinuation is planned. There are no criteria for modifying allocated interventions.

#### 3.6.4 Strategies to improve adherence to intervention protocol

To improve participant adherence to intervention protocols, the study investigator will provide participants with a detailed explanation of the experimental procedures, including how to inhale the sample. Before starting the experiment, the study investigator will also ask participants to practise the inhalation procedure (e.g. inhaling the sample with natural breathing, keeping the distance between the participant's nose and the bottle opening within 10 mm) to confirm participants' understanding of the procedure.

#### 3.6.5 Relevant concomitant care permitted or prohibited during the trial

Concomitant use of medicines (e.g. antidepressants, anxiolytics, hypnotics), herbal medicines, or health foods that may have anti-anxiety or anti-stress effects is not permitted. Participants are also instructed to keep to their regular diet on the experiment day, including alcohol consumption and physical activity before the study.

#### 3.6.6 Provisions for post-trial care

After the completion of this study, if an adverse event occurs that possibly is causally related to Shikuwasa essential oil inhalation, the principal investigator will provide the medical treatment deemed most appropriate for the participant. Other than such cases, since this is a low-risk study, there are no stipulations on post-trial care.

### 3.7 Outcomes

We set the following primary and secondary outcomes in this study:

#### 3.7.1 Primary outcome

- The tense arousal subscale scores of JUMACL

#### 3.7.2 Secondary outcomes

- Changes in miosis rate (autonomic nervous activity)
- Changes in peripheral skin temperature (autonomic nervous activity)
- Changes in cerebral blood flow (central nervous activity)
- Visual Analogue Scale (VAS) on individual preference and impression about Shikuwasa essential oils (participants in the control group will inhale Shikuwasa essential oils after completing the three experiments)
- Adverse events

Given the variability attributed to individual characteristics, this study adopts the changes from baseline as the outcomes for miosis rate, peripheral skin temperature, and cerebral blood flow. Baseline measurements were taken in odour-free air under controlled conditions, followed by measurements under intervention conditions. See the “Participant timeline” for detailed assessment time points, and the “Plans for assessment and collection of outcomes” for more detailed descriptions of each outcome.

### 3.8 Participant timeline

**Fig. 1:**
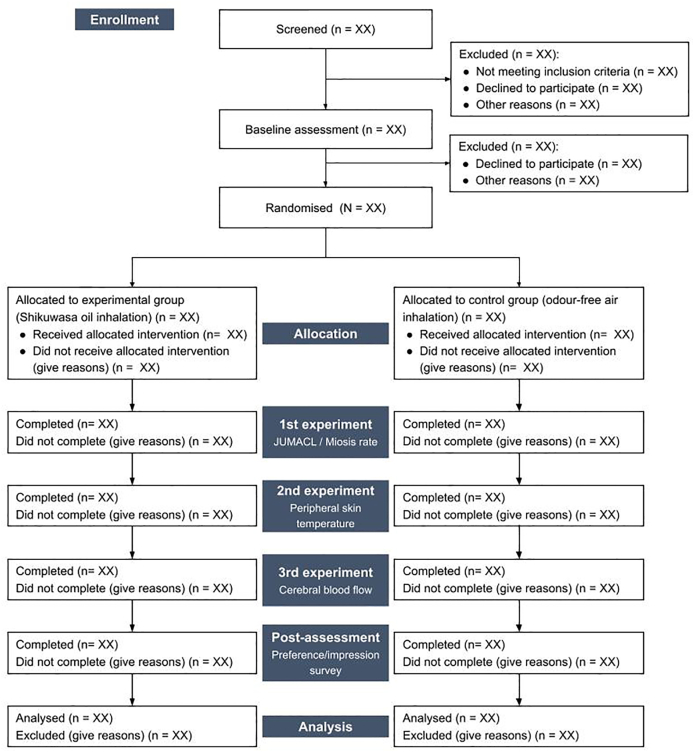
Flow diagram of the study.

The flow diagram of the study is presented in Fig. 1. Potential study participants will indicate their willingness to participate in the study by filling out the online form or by directly contacting the study office via email or phone call. Study investigators will then pre-screen potential participants using an eligibility checklist. If eligible according to the inclusion and exclusion criteria, the study office will invite potential participants to the study institution for informed consent. After obtaining informed consent and baseline participants’ information, participants will be randomly assigned to either the Shikuwasa essential oil inhalation group or the odour-free air inhalation group. Participants in both groups will then receive three experiments and post-assessment (Table 1). Same as in a previous study [30], we need to divide our experiments into three phases because these objective outcome measures (miosis rate, peripheral skin temperature, and cerebral blood flow) cannot be performed at the same time. Pre-assessment, allocation, experiment, and post-assessment will be conducted on the same day (approximately 1 h).

**Table 1:** Participant timeline.

	Screen	Pre-assessment, allocation, experiment, and post-assessment (1-h)					
		Pre-assessment	Allocation	Experiment^a^			Post-assessment
				1st	2nd	3rd	
Eligibility screen	X						
Demographics	X						
Informed consent		X					
JUMACL		X		X			
Allocation			X				
Miosis rate				X			
Peripheral skin temperature					X		
Cerebral blood flow^b^						X^b^	
Preference/impression survey							X
Adverse events				→			

Abbreviation: JUMACL, the Japanese version of the University of Wales Institute of Science and Technology Mood Adjective Checklist.

a Participants inhale allocated samples for 2 min in each experiment. A rest period separates the baseline measurement and task (inhaling allocated samples) for 2 min. The interval between each experiment is 10 min.

b Cerebral blood flow assessment involves continuous measurement of 1st rest (2 min), baseline (2 min), 2nd rest (2 min), task (inhaling allocated samples) (2 min), and 3rd rest (1 min).

### 3.9 Sample size

The sample size was calculated a priori. Assuming a group difference of 5 points (standard deviation = 5) in tense arousal scores of JUMACL at a post-inhalation time point based on unpublished preliminary data from our pilot study, 17 participants per group will provide >80 % power to detect a group difference in the primary outcome, using a two-sided, two-sample *t*-test at a 5 % level of significance. Considering that discontinuation or dropout occurs (loss of 3 participants per group), 20 participants are required per group, for a total sample size of 40 in this study.

### 3.10 Data collection and management

#### 3.10.1 Plans for assessment and collection of outcomes

Baseline demographic information, such as gender, date of birth, employment status, and some other information, will be collected at the pre-screening stage using an online self-report form. We will also assess participants' overall stress levels using the Stress-Check List 30-items (SCL30) [31,32] through an online self-report form. The SCL30 is a self-administered questionnaire consisting of 30 items designed to evaluate an individual's awareness of stress. Each item answered affirmatively is scored as 1 point, with total scores ranging from 0 to 30 points. A higher total score indicates a greater awareness of stress. The total scores are categorised into four levels: a score of 0–5 points indicates normal stress levels (low stress, no problem level), 6–10 points indicates mild stress (somewhat higher stress level), 11–20 points indicates moderate stress (a genuinely high-stress level), and over 20 points indicates severe stress (a high-stress level requiring professional examination or diagnosis).

The primary outcome is the tense arousal subscale score of JUMACL [28]. We chose this brief self-reported instrument as a primary outcome because, unlike some other self-reported instruments/questionnaires (e.g. Perceived Stress Scale, Depression Anxiety Stress Scale, and Lipp's Stress Symptoms Inventory), JUMACL and its original English language version [33] can assess immediate effects of interventions on mood and emotional status (i.e. level of arousal) and have been used in several studies using essential oils [34,35]. The JUMACL is a self-administered questionnaire comprising 20 items designed to assess an individual's current mood and emotional state (i.e. level of arousal). It consists of two subscales of “tension arousal”, evaluating feelings of tension and relaxation, and “energetic arousal”, evaluating vitality and energy, each consisting of 10 items in which respondents assess their current mood on a 4-point scale from “definitely yes” to “definitely not”. In this study, we focus on the tense arousal subscale scores (ranging from 10 to 40), with lower scores indicating greater relaxation. The reliability and validity data of the original English language version have been reported, including significant correlations with psychophysiological measures of arousal, suggesting its usefulness for measuring mood states [33]. Its Japanese version, JUMACL, has also been reported to exhibit adequate internal consistency [26], and the tense arousal subscale score has been demonstrated to be a good index of emotional stress among the Japanese university student sample [28].

To evaluate the relaxation effects of Shikuwasa essential oil inhalation (and odour-free air inhalation), we set different objective outcome measures (miosis rate, peripheral skin temperature, and cerebral blood flow) [17,30]. More specifically, we will use miosis rate and peripheral skin temperature for evaluating autonomic nervous activity, and cerebral blood flow (assessed using near-infrared spectroscopy) for evaluating central nervous activity.

The change in miosis rates will be determined with an Iriscoder Dual C-10641 (Hamamatsu Photonics & Co., Inc., Shizuoka, Japan). This general medical device allows for the precise measurement of changes in pupil diameter before and after inhalation of the samples, and increases in the miosis rate indicate parasympathetic dominance [30,36]. Miosis rate assessment is performed after 2 min of dark adaptation with goggles. After inhalation of essential oil or odour-free air, the pupil is briefly irradiated with a 10-s bright (100 cd/m2) red light for 0.2–1.0 s, and rapid-onset pupil constriction occurs due to light reflection. Thereafter, the pupil gradually redilates to a state of partial constriction in about 4 s [37]. This pupil diameter change is captured by a highly sensitive CCD camera, and the miosis rate is calculated. The miosis rate contraction is expressed as the ratio of the difference between the pupil diameter before light irradiation and the minimum pupil diameter after light irradiation to the pupil diameter before light irradiation. Measurements are taken on both eyes, and the average value is calculated and used as the change in measured value from baseline.

The change in peripheral skin temperature, which can be used as an indicator of sympathetic responses (increase in fingertip temperature represents decreased sympathetic nervous system activity) [38], will be measured using a skin thermistor (LT-ST08-12, Gram Co., Saitama, Japan). The temperature data will be processed using software developed in-house specifically for PC control and data storage purposes. To improve stability and operability during measurement because of the bare probe, we created a device that can simultaneously measure the fingertip and the centre of the forehead. The change in will be calculated from baseline.

Cerebral blood flow, which is changes in the concentration of oxy-Hb in the frontal cortex, will be quantified using a 16-channel near-infrared spectroscopy (NIRS) device (Spectratech OEG-16H, Spectratech Inc., Tokyo, Japan) [39]. A decrease in oxy-Hb concentration indicates a decrease in cerebral blood flow and reflects a decrease in cellular activity in the vicinity, suggesting sedation of the central nervous system [40]. After the sensor belt is placed on the head of the participant sitting in a resting position, calibration is performed, and after confirming that there are no problems, measurements are taken for 2 min before inhalation, 2 min during inhalation, and 2 min after inhalation. The change in mean oxy-Hb concentration for each channel will be calculated.

We will also investigate individual preferences/impressions regarding inhaled samples and adverse events. Subjective preference of the inhaled sample will be evaluated on 100-mm VAS (0 = do not like at all; 100 = like very much). Subjective impressions of the inhaled sample related to sample characteristics will be evaluated on the following 11 items on 100-mm VAS (0 = strongly disagree; 100 = strongly agree): “healing scent”, “elegant scent”, “relaxing scent”, “glamorous scent”, “fruity scent”, “sweet scent”, “refreshing scent”, “brilliant scent”, “scent of grass or trees”, “uplifting scent”, and “motivating scent”. All adverse events observed during the study will be coded and evaluated using the Common Terminology Criteria for Adverse Events (CTCAE) version 5.0, Japan Clinical Oncology Group version [41].

#### 3.10.2 Plans to promote participant retention and complete follow-up

Before entering the study, participants will receive a detailed explanation of the study, including possible benefits and the expected time required to complete the whole experimental procedure. In line with ethical and regulatory approval, a financial incentive (3000 yen) will be paid to each participant after completing the whole study procedure.

#### 3.10.3 Data management

All de-identified participant source data will be recorded on case report forms (CRFs), which will be centrally managed by the study office. To ensure high-quality data, data collected within the case report forms will be processed at the study office, using a licensed, automated, electronic data capture system (Viedoc™, Pharma Consulting Group, Uppsala, Sweden) which allows data to be entered, checked and validated. All essential documents and study records (both paper and electronic) will be retained for 5 years after study completion/discontinuation or 3 years after disclosure of the study results. After which time these documents/records will be securely destroyed.

#### 3.10.4 Confidentiality

All participants' data obtained in the study will be pseudo-anonymised and de-identified. The data will be kept under the participants’ study identification numbers throughout the study, which will be stored separately from the identifying information and from consent and assent forms. All written data will be stored in a locked cupboard in a locked room with access to the cupboard restricted to the study principal and co-investigators at the study office. All electronic data files will be password-protected and stored in a secured cloud-based electronic document management system, which will only be accessible by password and only by the study principal and co-investigators at the study office.

#### 3.10.5 Plans for collection, laboratory evaluation and storage of biological specimens for genetic or molecular analysis in this trial/future use

No biological specimens will be collected as part of this study.

### 3.11 Statistical methods

#### 3.11.1 Statistical methods for primary and secondary outcomes

In this study, we will adhere to the intention-to-treat principle to define the analysis populations. The population for efficacy analysis will be considered as the largest analysis set, while the population for safety analysis will be defined as the safety analysis set.

*Full Analysis Set (FAS):* Consists of all participants in the randomised set who are assigned to study treatment and had not received allocated intervention after randomisation, or those who have no post-inhalation data.

*Safety Analysis Set (SAF):* Consists of all participants in the full analysis set who received at least one inhalation of the sample. The participants will be classified based on the treatment actually received.

After the completion of the study, all data will be reviewed, and case discussion and data cleaning will be performed before locking the database for statistical analysis. The statistical analysis will be conducted by the principal investigator and supported by a designated researcher specialising in statistical analysis. The significance level for this analysis is set at a two-sided 5 %. Stata SE version 18.0 (Stata Corp., College Station, TX) will be used for statistical analyses.

For the participants' baseline demographic variables, summary statistics are constructed employing frequencies and proportions for categorical data, and means and standard deviations for continuous variables. The baseline variables between groups will be compared using Fisher's exact test (or chi-square test) or unpaired t-tests (Welch's *t*-test for unequal variances or Mann-Whitney *U* test for non-normal data).

The effects of inhaled samples on the primary outcome (tense arousal subscale scores of JUMACL) and the secondary outcomes (excluding adverse events: miosis rate, peripheral skin temperature, cerebral blood flow, and individual preference/impression on VAS) will be examined through between-group comparisons using unpaired t-tests (Welch's *t*-test for unequal variances or Mann-Whitney *U* test for non-normal data).

The analyses of adverse events will be descriptive in nature, and an overall summary of adverse events (e.g. the frequency of participants with adverse events) in each group will be presented. When between-group comparisons are needed, Fisher's exact test (or chi-square test) or unpaired t-tests (Welch's *t*-test for unequal variances or Mann-Whitney *U* test for non-normal data) will be utilised.

#### 3.11.2 Interim analyses

Since there are no anticipated problems that are detrimental to the participant, an interim analysis is not warranted.

#### 3.11.3 Methods for additional analyses (eg, subgroup analyses)

For subgroup analysis, participants will be divided into two groups by median baseline SCL30 score (high score group and low score group), and each group will be analysed for the primary and secondary outcomes. Missing data will be analysed to the maximum extent possible, although secondary outcomes (excluding adverse events) may be missing due to problems with the measurement equipment or other reasons.

#### 3.11.4 Methods in analysis to handle protocol non-adherence and any statistical methods to handle missing data

Missing data will be minimised by careful study conduct and data management; thus, it is anticipated that missing data will be minimal. Missing data will be described with reasons given where available; the number and percentage of individuals in the missing category will be clearly reported. We do not plan to impute missing values, but in the event of substantial missing data, may consider the use of any method of imputation within sensitivity analysis. More specifically, if missing data occurs in more than 5 participants, a sensitivity analysis method will be planned in advance before fixing the dataset for analyses.

#### 3.11.5 Plans to give access to the full protocol, participant level-data and statistical code

The study information is available at the University Hospital Medical Information Network Clinical.

Trials Registry (UMIN-CTR), UMIN000053914. The Japanese version of the complete study protocol is available upon reasonable request to the corresponding author. Anonymised data from this paper are also available for research purposes on request to the corresponding author. Broader public data sharing for this study has been restricted by the Research Ethics Committee of the Faculty of Medicine, University of Miyazaki because the consent obtained from the participants does not cover the unlimited public sharing of the data.

### 3.12 Oversight and monitoring

#### 3.12.1 Composition of the coordinating centre and trial steering committee

This study does not have a coordinating centre or trial steering committee. The principal investigator (YA) is responsible for the overall aspects of the study. The principal and co-investigators at the study office meet every week to oversee the progress of the study and the completion of tasks, and to review necessary changes to the protocol to facilitate the smooth running of the study.

#### 3.12.2 Composition of the data monitoring committee, its role and reporting structure

Due to the low-risk nature of the intervention, the short duration of the study, and the fact that interim analyses may not provide necessarily informative results given the nature of the study, the establishment of a data monitoring committee is not considered necessary.

#### 3.12.3 Adverse event reporting and harms

All adverse events and severe adverse events occurring during the study will be recorded in CRF. When an adverse event occurs, the study investigators will take all necessary and appropriate measures to ensure the safety of the participant. Based on the Japanese government's “Ethical Guidelines for Medical and Health Research Involving Human Subjects”, adverse events that result in any of the following events will be defined as serious: 1) fatal events, 2) life-threatening events, 3) events that require hospitalisation, 4) events that result in persistent or significant disability or incapacity, and 5) events that cause a congenital anomaly or birth defect. When a serious adverse event occurs, regardless of the causal relationship with the study intervention, the study principal investigator must report it promptly to its own Research Ethics Committee and the institution's representative in accordance with the policies and procedures of the institution.

#### 3.12.4 Frequency and plans for auditing trial conduct

No audit is planned for the conduct of the study as this is a non-invasive intervention study.

### 3.13 Patient and public involvement

There is no patient and public involvement in the design, conduct, reporting or dissemination plans of this study.

## 4 Ethics and dissemination

### 4.1 Ethics approval and consent to participate

The study protocol has been reviewed and approved by the Research Ethics Committee of the Faculty of Medicine, University of Miyazaki (reference no: I-0074). Written informed consent to participate in this study will be taken from all participants.

#### 4.1.1 Informed consent

Study investigators will pre-screen potential participants using an eligibility checklist based on information the participants fill out in the online form. If eligible according to the inclusion and exclusion criteria, the study office will invite potential participants to the Faculty of Medicine, University of Miyazaki for informed consent. Study investigators will read the informed consent form together with potential participants. Key information about the aims of the study, procedures, expected risks and benefits, protection of personal information, the right of withdrawal, and the voluntary nature of participation will be reviewed. Finally, participants will decide whether to participate in the study. All participants will sign informed consent before initiating the study procedure.

#### 4.1.2 Additional consent provisions for collection and use of participant data and biological specimens

No additional participant data or biological specimens will be collected.

### 4.2 Dissemination plans

We will follow the Consolidated Standards of Reporting Trials (CONSORT) guidelines for reporting the results of this study [42]. The findings of this study will be disseminated to academic and professional audiences via publications in peer-reviewed journals and presentations at academic conferences, and to the broader public via public talks and media/press releases. All findings from the study, whether negative or positive, will be reported.

### 4.3 Consent for publication

Participants will be informed that the results and data from this study will be published in anonymised form in scientific journals, registries, and an online repository before they sign the consent form.

### 4.4 Plans for communicating important protocol amendments to relevant parties (e.g. trial participants, ethical committees)

All protocol amendments will follow the Research Ethics Committee's revision process and will be approved prior to their implementation. Where necessary, the study investigators will inform study participants of important protocol modifications. All relevant information in the trial registration on UMIN-CTR will also be updated.

## 5 Discussion

The purpose of this study is to examine the short-term relaxation effects of Shikuwasa essential oil inhalation compared to odour-free air inhalation in young female adults through a randomised controlled trial. This exploration contributes to the broader understanding of Shikuwasa essential oil and its applications in stress management and relaxation, highlighting the cultural and physiological significance of these natural remedies.

The recent trend of Generation X and millennials for health and awareness of the benefits of natural medicine generates a high demand for essential oil products among general citizens [43]. Furthermore, aromatherapy using citrus is utilised to relieve many symptoms, such as body pain, nausea, vomiting, anxiety, depression, stress, insomnia, etc. [44]. Thus, findings from this study will provide meaningful insights into this growing health consciousness. If the psychological and physiological effects of citrus essential oils (incl. Shikuwasa) can be elucidated, essential oils may provide both an effective means to relaxation in today's stressful society and also increase the value and demand for citrus fruits.

It should be noted that some of the outcomes used in this study (e.g. JUMACL and VAS) are subjective. However, our study incorporates various objective outcome measurements (i.e. miosis rate, peripheral skin temperature, and cerebral blood flow) that allow us to speculate on participants’ psychological/physiological states; this will supplement the results of subjective outcomes and strengthen our findings.

There are three major limitations to this study. First, our study purposefully devises our experiment into three phases due to the methods of objective outcome measurements, so the repeated experiments might affect the results of the latter two experimental phases (e.g. the participants will get used to the inhalation sample). Second, our study focuses on the short-term effects of Shikuwasa essential oil inhalation among young Japanese female adults; so, its medium and long-term effects, and its effects on other age, gender, and race/ethnic groups cannot be evaluated. Finally, this study employed a non-active control condition (i.e. not an active control); so we cannot evaluate the superiority or non-inferiority of relaxation effects of Shikuwasa essential oil inhalation against other essential oils (e.g. lavender) or other citrus essential oils (e.g. orange, Yuzu, and Iyokan) that have already been shown to be effective [11,17,45,46]. However, the latter two limitations can lead to future research. If the current proposed study can show that inhalation of Shikuwasa essential oil has short-term relaxation effects on young Japanese female adults, the next step might be to examine its medium and long-term effects, and whether the relaxation effects of Shikuwasa essential oil inhalation can be translated into different age, gender, and race/ethnic groups. It is also worth investigating in future research which specific components in Shikuwasa essential oil contribute to its relaxation effects. Limonene, a key component known for its relaxation properties [9], is one of the predominant compounds in Shikuwasa (along with γ-terpinene), but it is not the primary aroma component in the essential oil [20].

## Trial status

Protocol version 1.0, 2024-03-21. The overall study is planned to begin in March 2024, and is estimated to be completed in March 2026. Recruitment is planned to begin in April 2024, and to be completed in June 2024.

## Funding

This study will be funded by a cooperative research grant provided by Ogawa & Co., Ltd. A joint research agreement between Ogawa & Co., Ltd. and the study team at the University of Miyazaki was signed. Ogawa & Co., Ltd. provided the Shikuwasa essential oils and objective measurement devices (Iriscoder Dual C-10641, LT-ST08-12, and Spectratech OEG-16H) used for the study free of charge, and the study co-investigators from the company supported the design of the study. However, recruitment, experiments, management, collection, analysis, and the decision to submit the report for publication are entirely independent of Ogawa & Co., Ltd.

## Availability of data and materials

The study principal and co-investigators at the study office will have access to the final study dataset.

## CRediT authorship contribution statement

**Fumitake Yamaguchi:** Writing – review & editing, Writing – original draft, Methodology. **Naoki Yoshinaga:** Writing – review & editing, Writing – original draft, Methodology. **Miho Kuroki:** Writing – review & editing, Methodology. **Rie Nakasone:** Writing – review & editing, Methodology. **Hisanori Kenmotsu:** Writing – review & editing, Methodology. **Toshio Ueno:** Writing – review & editing, Methodology. **Yukihiro Yada:** Writing – review & editing, Methodology. **Michikazu Nakai:** Writing – review & editing, Formal analysis. **Yasuji Arimura:** Writing – review & editing, Project administration, Formal analysis.

## Declaration of competing interest

The authors declare the following financial interests/personal relationships which may be considered as potential competing interests: RN, KH, and UT are employees of Ogawa & Co., Ltd. YY received an academic consulting fee from Ogawa & Co., Ltd. YA will be hired by a cooperative research grant provided by Ogawa & Co., Ltd. during the study. The other authors declare that they have no competing interests.
